# A Note on the Tracing of Herbage Contribution to Grazing Sheep Diet Using Milk and Feces Biomarkers

**DOI:** 10.3389/fvets.2021.623784

**Published:** 2021-02-19

**Authors:** Giovanni Molle, Andrea Cabiddu, Mauro Decandia, Marco Acciaro, Giuseppe Scanu, Margherita Addis, Myriam Fiori, Marco Caredda

**Affiliations:** Agris Sardegna, Olmedo, Italy

**Keywords:** dairy sheep, nutrition, traceability, alkanes, fatty acids, pasture, chemometrics, authentication

## Abstract

Milk from grazing ruminants is usually rich in beneficial components for human health, but distinguishing milks sourced from grazing is difficult, and this hinders the valuing of the grazing benefit. This study aimed at evaluating the ability of milk biomarkers (1) to trace milks sourced from sheep submitted to different access times (ATs) to pasture and (2) to estimate sheep herbage dry matter intake (HDMI, g DM ewe^−1^ d^−1^) and herbage percentage (HP, % DM) in sheep diet. Animal data derive from a published experiment in which six replicated groups of mid-lactation Sarda sheep had ATs of 2, 4, or 6 h d^−1^ to a ryegrass pasture. Sheep HDMI and HP of each group were measured on four dates in April 2013. Group milk was sampled, and milk fatty acids (FAs) and n-alkanes were determined by gas chromatography. The latter markers were also measured in feces samples bulked by group. The data (*N* = 24 records) were submitted to Linear Discriminant Analysis (LDA) aimed at distinguishing the AT to pasture based on biomarkers previously selected by Genetic Algorithms (GA). Partial Least Square Regression (PLSR) models were used to estimate HDMI and HP using biomarkers selected by GA. Based on one milk alkane and six milk FAs as biomarkers, estimates of the AT using GA-LDA were 95.8% accurate. The estimation of HDMI by GA-PLSR based on five milk FAs was moderately precise [explained variance = 75.2%; percentage of the residual mean square error of cross-validation over the mean value (RMSECV%) = 15.0%]. The estimation of HP by GA-PLSR based on 1 milk alkane and 10 FAs was precise (explained variance = 80.8%; RMSECV% = 7.4%). To conclude, these preliminary results suggest that milks sourced from sheep flocks with AT to pasture differentiated by 2 h in the range 2–6 h d^−1^ can be precisely discriminated using milk biomarkers. The contribution of herbage to sheep diet can also be precisely estimated.

## Introduction

Grazing delivers high-quality ruminant products at low cost as compared with stall feeding, as highlighted by recent reviews (1, 2). Positive implications were often found on these products, with reference to their nutritional and health value as well as technological and sensory attributes, such as texture, oxidative stability, and flavor (1) with few exceptions [e.g., (3)]. Moreover, consumers perceive pasture-based livestock systems as more friendly for both environment and animal welfare than housed systems (4).

Unfortunately, pasture availability is seasonal and often scarce in many grazing areas, such as the Mediterranean regions; hence, supplementation is necessary, at least for part of the pasture growth cycle. For this reason, part-time grazing (PTG), i.e., a time-restricted allocation to pasture, is often implemented in Mediterranean dairy sheep systems. This technique has revealed several beneficial implications compared with stall feeding and 24-h grazing, among them, the saving of herbage when herbage growth is low and a more balanced diet (5). Moreover, a moderate restriction of access time (AT) to pasture (6 h/d) can suffice to reach levels of beneficial FA in milk as high as those achieved with longer allocations (9 h/d), as shown in dairy cattle (6).

In order to increase the value of grazing in the sheep supply chain, tracing the milk back to the feeding system is fundamental. Tracing can be based on biomarkers, such as milk FA and fecal and milk concentration of n-alkanes, which are long-chain hydrocarbons contained in plant epicuticular or waxes (7). N-alkanes, particularly those with a short chain, can be uptaken by the gut mucosa to some extent and pass without changes in ruminant milk (8). In fact, milk alkane profile was successfully used to discriminate cheeses derived from cows grazing pastures with different botanical compositions (9).

Despite the growing body of knowledge on biomarkers of dairy products, the impact of these findings is still limited, because milk FAs and alkanes composition can possibly fail to discriminate milks coming from semi-intensive systems, where PTG is practiced (10).

This paper is an outcome of a wider research program undertaken at Agris Sardegna in 2013–2016 for evaluating the impact of PTG of dairy ewes on their ingestive behavior and milk production (5, 11). This specific study is aimed at evaluating the ability of milk FAs and n-alkanes measured in both milk and feces 1) to trace bulk milks sourced from sheep submitted to PTG with different ATs to pasture and 2) to estimate sheep herbage dry matter intake (HDMI, g DM ewe^−1^ d^−1^) and herbage percentage (HP, % DM) in sheep diet. Since Genetic Algorithms (GA) had already been successfully used to select the informative variables for the estimation of the sheep milk fatty acids (FAs) by mid-infrared spectroscopy (12) and in the selection of the FAs able to trace the geographical origin of sheep milk (13), we investigated their use to the aims of the present work.

## Materials and Methods

The animal protocol and implemented procedures were in accordance with the ethical guidelines in force at Agris, in compliance with the EU directive 86/609/EC and the recommendation of the EU Commission 2007/526/EC.

The study was conducted at the Bonassai research station, north-western Sardinia (40°N, 8°E, 32 m a.s.l.). The whole experiment lasted from February to April 2013, but, for the purpose of this work, the experimental period ranges from 10 to 23 April. This short period was chosen to represent the spring period in which neither availability nor quality of grazed grass limits animal performance. The climate is Mediterranean with a long-term (1995–2013) average annual rainfall of 568 mm. A randomized block design was adopted, with two replicates per treatment. Pasture consisted of 1.5 ha of Italian ryegrass (*Lolium multiflorum* Lam. cv. Teanna). The area was split into two blocks of 7,500 m^2^ each, which were in turn divided into three experimental plots (*n* = 6 plots in total) randomly allocated to the treatments. Each plot was then divided by electric fences into four sub-plots of 625 m^2^ each, which were rotationally grazed, with 7 days of occupation per sub-plot and a recovery period of 21 days.

Six groups of six ewes each, all belonging to Sarda breed, lambed in autumn (November–December) and at the mid-lactation stage (mean ± standard deviation 76 ± 14 days in milk) at the beginning of the experiment, were balanced for sheep age (3.7 ± 1.2 years), pre-experimental milk yield (1,449 ± 206 g/d), and body weight (42.5 ± 4.0 kg) and randomly assigned to the experimental plots. The ewes were machine milked twice daily at 07:00 and 15:00 h. After morning milking, the groups were carried on a trailer to the plots where they spent the scheduled time. Treatments were three different levels of ATs to pasture: 2 (08:00–10:00), 4 (08:00–12:00), and 6 h/d (08:00–14:00). During the remaining daytime, the ewe groups were kept indoors in separate pens. Supplementation consisted for all ewes of pelleted concentrate (400 g/head day split in two meals at milkings), lupin seed (300 g/head day) at pasture turnout, and ryegrass hay (700 g/head day) overnight. The flat supplementation rate was set in order to meet 100% of energy requirement of the 4 h/d treatment and 100% metabolic protein requirements of 2 h/d treatment. For details on pasture establishment and animal management, refer to Molle et al. (11).

### Measurements and Samplings

Supplements intake was measured at group level, weighing the offer and the refusals either after each meal (concentrates) or after 24 h (hay). On four occasions during the experimental period (test days), short-term herbage intake rate was measured on three ewes per group using the double-weighing technique as detailed in depth by Molle et al. (5). The day after each test day, individual milk yield was measured and milk sampled for determining milk fat, protein, and lactose contents (MilkoScan FT+; Foss Electric, Hillerød, Denmark). Bulk milk samples of each group of ewes for each treatment were also collected for milk FA and n-alkane determinations. Samples of supplements and hand-plucked samples of ryegrass potentially eaten by the sheep were taken on the intake measurement days. Moreover, feces were also individually grab-sampled from each ewe tested for intake measurement after each milking on the days of milk recording. All these samples were immediately frozen and then freeze-dried prior to analysis. The sample of feces was bulked per group before chemical determinations.

### Chemical Analysis

The FA composition of the milk samples was determined as reported in Caredda et al. (12). The FA content of herbage and supplements was determined according to Addis et al. (14). Supplement FA composition was measured on a composite sample per feedstuff. The n-alkane analysis of herbage and feces followed the protocol by Dove and Mayes (15). A similar protocol was implemented for milk alkane determination. Milk, feeds, and feces alkane analytical method and gas chromatographic conditions are reported as Supplementary Material. Individual n-alkanes from C23 to C36 were identified by the comparison of the retention time of a standard mixture of pure components. Furthermore, indices were calculated with reference to n-alkane, such as the ratio between the concentrations of adjacent alkanes with carbon chain length ranging between C27 and C33 (C27/C25, C29/C27, C31/C29, and C33/C31). Feedstuff samples were also submitted to analyses for the evaluation of their nutritional value according to the methods detailed by Molle et al. (5). Data on feedstuff nutritional, FA, and n-alkane compositions are reported in Supplementary Table 1.

### Statistical Analysis

The database was constituted of *N* = 24 group records (3 treatments × 2 replicates × 4 dates), inclusive of treatment (AT, h/day), replicate (1, 2), date, and all the measured biomarkers (alkane in feces and milk and FA in milk and their classes and indices). Since the two replicates actually consisted of two different groups of ewes, the obtained milk samples were not considered replicates from a chemometric point of view but different samples belonging to the same treatment category. Means with standard deviation of the distribution and ranges of the data across the experimental period are shown in Table 1. Correlation analysis was used to explore the relationships among variables.

**Table 1:** Mean ± standard deviation of the distribution (SD) and range of the variables under study.

		**Mean ± SD**	**Range**	
			**Max**	**Min**
Herbage intake	g DM/d	1,113 ± 342	1,768	580
Total intake	g DM/d	2,268 ± 337	2,947	1,686
Herbage in diet	% DM	47.9 ± 8.2	60.00	34.42
**n-Alkane in feces**				
C24	mg/kg DM	1.0 ± 1.7	6.74	0.00
C25	mg/kg DM	16.6 ± 2.7	24.69	12.98
C26	mg/kg DM	2.4 ± 1.0	6.07	1.58
C27	mg/kg DM	54.3 ± 4.6	61.93	45.25
C28	mg/kg DM	24.3 ± 9.1	44.35	10.01
C29	mg/kg DM	274.9 ± 34.3	350.73	213.83
C30	mg/kg DM	16.3 ± 2.3	21.52	12.75
C31	mg/kg DM	446.9 ± 60.4	565.75	332.93
C32	mg/kg DM	8.6 ± 2.7	17.19	5.02
C33	mg/kg DM	84.7 ± 16.3	115.00	56.40
C35	mg/kg DM	3.4 ± 2.2	6.18	0.00
R2725F		3.32 ± 0.45	4.40	2.41
Ratio 29/27		5.0 ± 0.4	5.66	4.37
Ratio 31/29		1.62 ± 0.06	1.77	1.54
Ratio 33/31		0.19 ± 0.03	0.27	0.15
**n-Alkane in milk**				
C24	mg/L	1.1 ± 0.4	1.89	0.00
C25	mg/L	3.0 ± 1.1	5.31	1.01
C26	mg/L	1.2 ± 0.4	1.90	0.00
				
C27	mg/L	4.0 ± 0.5	4.81	3.12
C29	mg/L	7.6 ± 0.8	8.74	6.36
C30	mg/L	0.9 ± 1.2	5.80	0.00
C31	mg/L	4.7 ± 1.5	6.24	0.00
Ratio 27/25		1.5 ± 0.7	3.55	0.87
Ratio 29/27		1.9 ± 0.2	2.20	1.62
Ratio 31/29		0.6 ± 0.2	0.78	0.00
**Fatty acids in milk**				
C4:0	% FAME	3.96 ± 0.15	4.30	3.71
C6:0	% FAME	2.80.1	2.99	2.49
C7:0	% FAME	0.03 ± 0.01	0.05	0.02
C8:0	% FAME	2.33 ± 0.15	2.60	1.99
C10:0	% FAME	6.7 ± 0.5	7.88	5.76
C11:0	% FAME	0.34 ± 0.03	0.44	0.27
C12:0	% FAME	3.7 ± 0.3	4.44	3.22
C13:0 *iso*	% FAME	0.035 ± 0.005	0.05	0.03
C13:0 *anteiso*	% FAME	0.046 ± 0.004	0.06	0.04
C14:0 *iso*	% FAME	0.13 ± 0.01	0.16	0.11
C14:0	% FAME	11.2 ± 0.6	12.77	10.40
C15:0 *iso*	% FAME	0.40 ± 0.04	0.46	0.32
C15:0 *anteiso*	% FAME	0.65 ± 0.04	0.76	0.58
C14:1 9c	% FAME	0.20 ± 0.02	0.25	0.15
C15:0	% FAME	1.18 ± 0.05	1.31	1.10
C16:0 *iso*	% FAME	0.33 ± 0.03	0.41	0.28
C16:0	% FAME	25.0 ± 1.1	27.21	22.59
C17:0 *iso*	% FAME	0.58 ± 0.05	0.67	0.49
C16:1 7c	% FAME	0.28 ± 0.02	0.33	0.25
C17:0 *anteiso*	% FAME	0.62 ± 0.04	0.71	0.54
C16:1 9c	% FAME	0.75 ± 0.07	0.90	0.62
C17:0	% FAME	0.69 ± 0.05	0.80	0.58
C17:1 10c	% FAME	0.18 ± 0.02	0.20	0.16
C18:0	% FAME	10.2 ± 0.7	11.75	8.71
C18:1 4t	% FAME	0.013 ± 0.003	0.02	0.01
C18:1 5t	% FAME	0.013 ± 0.004	0.02	0.00
C18:1 6t ÷ 8t	% FAME	0.18 ± 0.02	0.23	0.15
C18:1 9t	% FAME	0.20 ± 0.02	0.24	0.17
C18:1 10t	% FAME	0.30 ± 0.05	0.43	0.23
C18:1 11t	% FAME	1.2 ± 0.2	1.73	0.92
C18:1 12t	% FAME	0.35 ± 0.05	0.42	0.26
C18:1 13t ÷ 14t	% FAME	0.9 ± 0.1	1.22	0.67
C18:1 9c	% FAME	17.5 ± 0.9	19.32	15.81
C18:1 15t + 10C	% FAME	0.6 ± 0.2	1.04	0.40
C18:1 11c	% FAME	0.28 ± 0.02	0.31	0.24
C18:1 12c	% FAME	0.15 ± 0.03	0.20	0.11
C18:1 13c	% FAME	0.06 ± 0.01	0.07	0.05
C18:1 14c + 16t	% FAME	0.48 ± 0.05	0.59	0.38
C18:2 9t, 12t	% FAME	0.03 ± 0.01	0.05	0.02
C18:2 9c, 13t	% FAME	0.39 ± 0.05	0.49	0.30
C18:2 9c, 12t	% FAME	0.16 ± 0.02	0.18	0.13
C18:1 16c	% FAME	0.13 ± 0.02	0.17	0.11
C18:2 9t, 12c	% FAME	0.024 ± 0.004	0.04	0.02
C18:2 11t, 15c	% FAME	0.21 ± 0.05	0.31	0.14
C18:2 9c, 12c	% FAME	1.8 ± 0.2	2.08	1.55
C18:2 9c, 15c	% FAME	0.03 ± 0.01	0.04	0.02
C20:0	% FAME	0.28 ± 0.02	0.35	0.25
C18:3 6c, 9c, 12c	% FAME	0.05 ± 0.01	0.07	0.03
C20:1 9c	% FAME	0.02 ± 0.01	0.04	0.01
C20:1 11c	% FAME	0.05 ± 0.01	0.09	0.04
C18:3 9c, 12c, 15c	% FAME	0.60 ± 0.08	0.82	0.47
CLA 9c, 11t	% FAME	0.60 ± 0.06	0.72	0.48
CLA 9t, 11c	% FAME	0.08 ± 0.01	0.10	0.07
CLA 11t, 13c	% FAME	0.014 ± 0.003	0.02	0.01
CLA 12t,14t	% FAME	0.03 ± 0.01	0.04	0.01
CLA 11t, 13t	% FAME	0.03 ± 0.01	0.05	0.02
CLA 9t, 11t	% FAME	0.022 ± 0.003	0.03	0.02
C20:2 11c, 14c	% FAME	0.03 ± 0.01	0.05	0.01
C20:3 5c, 8c, 11c	% FAME	0.21 ± 0.02	0.27	0.17
C22:0	% FAME	0.023 ± 0.002	0.03	0.02
C20:3 8c, 11c, 14c	% FAME	0.03 ± 0.02	0.11	0.01
C20:3 11c, 14c, 17c	% FAME	0.01 ± 0.01	0.04	0.00
C20:4 5c, 8c, 11c, 14c	% FAME	0.17 ± 0.01	0.19	0.15
C23:0	% FAME	0.06 ± 0.01	0.08	0.04
C24:0	% FAME	0.05 ± 0.01	0.06	0.03
C20:5 5c, 8c, 11c, 14c, 17c	% FAME	0.05 ± 0.01	0.06	0.03
C26:0	% FAME	0.033 ± 0.004	0.04	0.02
C22:5 7c, 10c, 13c, 16c, 19c	% FAME	0.10 ± 0.01	0.13	0.09
C22:6 4c, 7c, 10c, 13c, 16c, 19c	% FAME	0.04 ± 0.01	0.05	0.03
Saturated FA	% FAME	71.4 ± 1.2	74.51	69.83
Unsaturated FA	% FAME	28.6 ± 1.2	30.17	25.49
Monounsaturated FA	% FAME	24.0 ± 1	25.36	21.41
Polyunsaturated FA	% FAME	4.7 ± 0.3	5.25	4.08
n−6 FA	% FAME	2.3 ± 0.2	2.59	1.97
n−3 FA	% FAME	0.96 ± 0.09	1.21	0.80
Ratio n3/n6		0.42 ± 0.04	0.49	0.35

Linear Discriminant Analysis (LDA) was used for classifying the samples coming from sheep submitted to different feeding regimens, and the resulting confusion matrix was evaluated both in terms of accuracy (calculated as the average of the percentages of correct predictions of each category) and in terms of Cohen's kappa (κ) that takes into account the possibility of correct classifications occurring by chance (16). The Mahalanobis distance between each sample and the centroids of the three treatment groups were also evaluated. Partial Least Square Regression (PLSR) was used to predict sheep HDMI and HP in sheep diet. The models were built both using all the identified biomarkers in milk and feces as predictors and using the informative biomarkers selected, separately for each dependent variable, by means of GA (17, 18). The validation of the models was achieved through the cross-validation approach. LDA and PLSR were run on the CAT (Chemometric Agile Tool) software, developed by the Group of Chemometrics of the Division of Analytical Chemistry of the Italian Chemical Society, freely downloadable from the site gruppochemiometria.it.

## Results

Although comparing the effects of treatment on performance goes beyond the scope of this study, it is worth noting in Supplementary Table 1 that the herbage contents of crude protein (CP, 137–142 g/kg DM) and neutral detergent fiber (NDF, 443–460 g/kg DM) showed a narrow range between groups, differently from herbage intake [mean ± SD, 718 ± 105 g DM (2 h/d), 1,248 ± 227 g DM (4 h/d), and 1,372 ± 216 g DM (6 h/d)], total intake [1,891 ± 128 g DM (2 h/d), 2,380 ± 217 (4 h/d), and 2,532 ± 230 (6 h/d)], percentage of herbage in the diet [38 ± 3% DM (2 h/d), 52 ± 5% DM (4 h/d), and 54 ± 4% DM (6 h/d)], and milk yield [921 ± 130 g (2 h/d), 904 ± 109 g (4 h/d), and 1,068 ± 123 g (6 h/d)]. In the whole experiment, herbage and total intake as well as milk yield were higher in 6 h/d than in the other treatment groups, being the 4 h/d group means intermediate (11).

Using all the variables as predictors, the LDA performance was not accurate in estimating the AT to pasture, with only 25% of correct predictions (κ = −0.125). In contrast, the GA-LDA showed high accuracy (95.8% of correct classification and a κ = 0.9375 that indicates an almost perfect agreement, being close to 1) using one milk n-alkane and six milk FAs as biomarkers (Table 2). The selected biomarkers were the n-alkane C24 and the following milk FAs C13:0 *iso*, C14:0, C16:1 7c, C18:1 11t, CLA 11t 13c, and C22:5 7c 10c 13c 16c 19c. Only one milk sample belonging to the 4 h/d group was misclassified as 2 h/d.

**Table 2:** Confusion matrix obtained by GA-LDA in tracing the diet of lactating ewe groups submitted to different part-time grazing regimens differing for daily access time (2, 4, and 6 h/d).

	**2 h/d**	**4 h/d**	**6 h/d**
2 h/d	8	0	0
4 h/d	1	7	0
6 h/d	0	0	8

**Figure 1:**
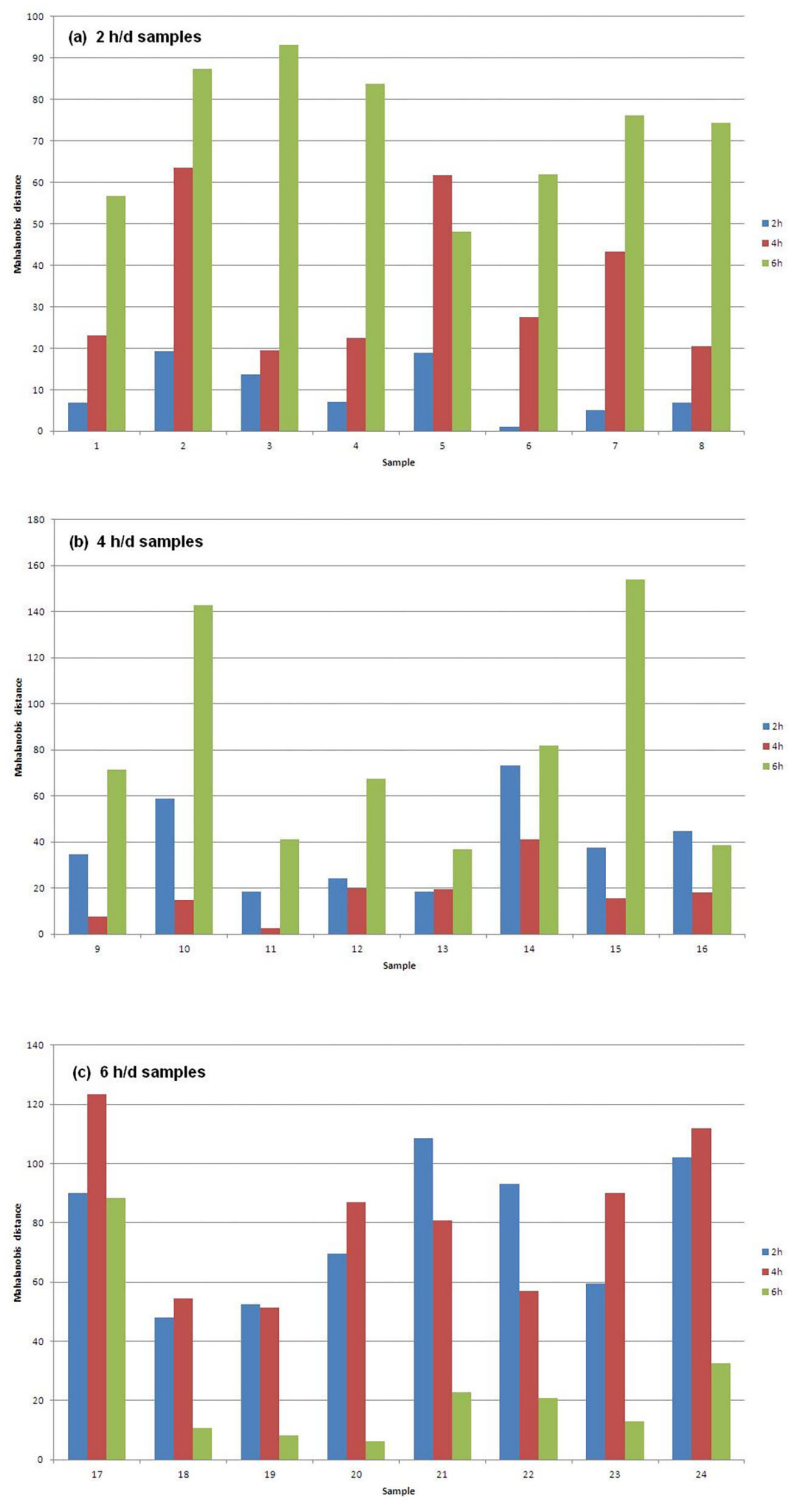
Mahalanobis distances of the **(a)** 2, **(b)** 4, and **(c)** 6 h/d samples, from the centroids of the three treatment groups of samples.

Figure 1 shows the Mahalanobis distances of each sample from the centroids of the three groups of samples that differ for AT to pasture. In particular, Figure 1a plots the 2 h/d samples, Figure 1b plots the 4 h/d samples, and Figure 1c plots the 6 h/d samples. It can be seen that every sample is well classified since its distance to the centroid of the group it belongs to is way shorter than the distances to the centroids of the other groups. The exception is the only misclassified sample (#13) in Figure 1b (4 h/d group), which is only slightly further from the centroid of its true group than from that of the group it has been assigned to (2 h/d), meaning that the only error in the assignment has not been great.

**Figure 2:**
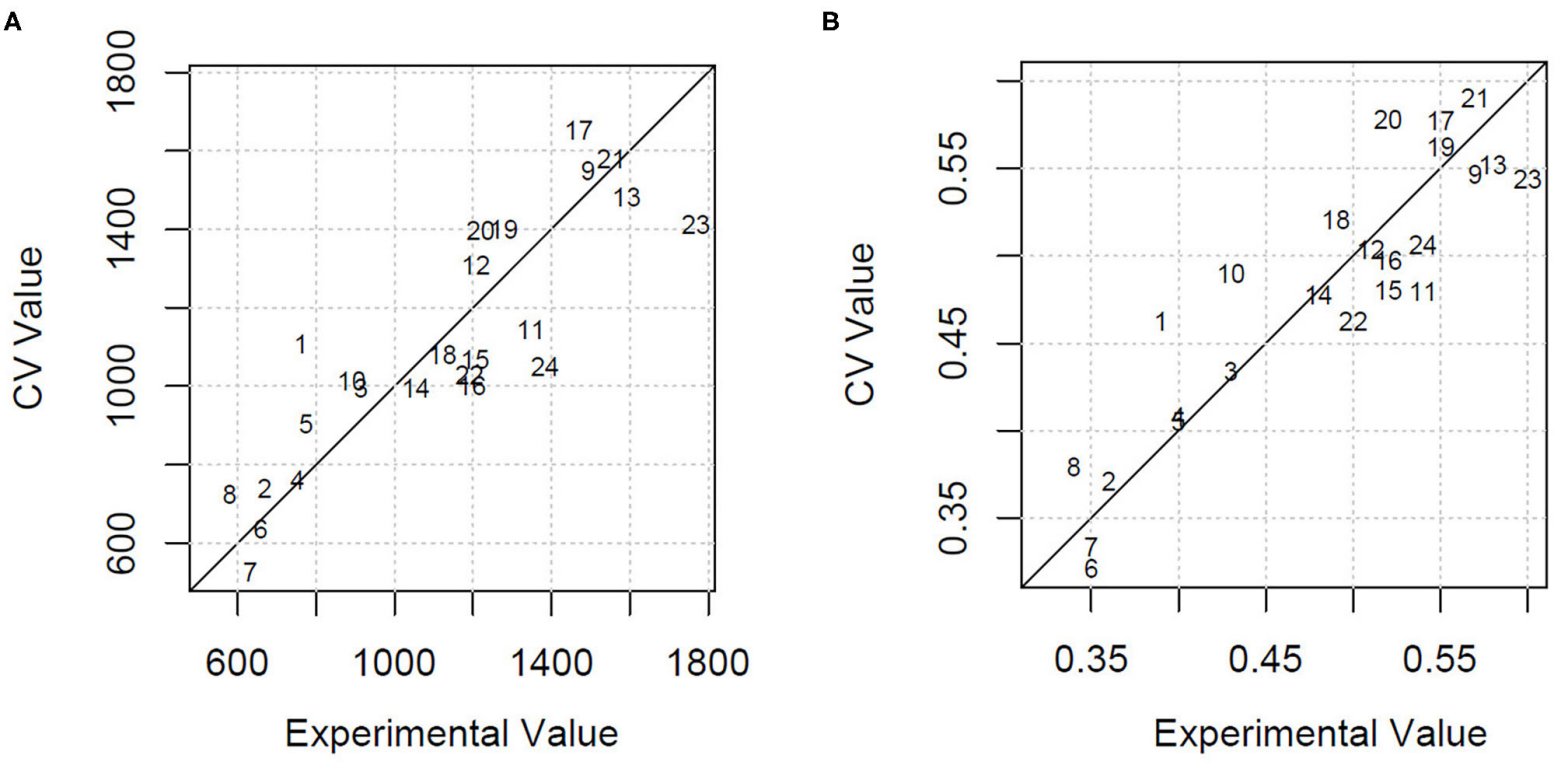
**(A)** Cross-validated (CV) vs. experimental value plot obtained by the GA-PLSR model to estimate the herbage intake of lactating ewe groups submitted to different part-time grazing regimens differing for daily access time (2, 4, and 6 h/d). **(B)** Cross-validated (CV) vs. experimental value plot obtained by the GA-PLSR model to estimate the herbage percentage in the diet of lactating ewe groups submitted to different part-time grazing regimens differing for daily access time (2, 4, and 6 h/d).

Using all the variables as predictors, the estimation of HDMI was moderately precise, with a percentage of the residual mean square error of cross-validation over the mean value (RMSECV%) equal to 22.1% and an explained variance of 46.3%. GA applied to PLSR selected only four milk FAs (C13:0 *iso*, C20:0, CLA 11t 13t, C20:5 5c 8c 11c 14c 17c) and the ratio n3/n6. Using these few variables, the HDMI estimation improved markedly, with a lowering of the RMSECV% to 15.0% and an increase of the explained variance to 75.2%. Figure 2A shows the cross-validated vs. the experimental value plot obtained by the model built with the selected variables.

The model built by PLSR using all the variables for the estimation of HP explained 56.5% of variance and predicted the HP values with an RMSECV% of 11.1%. This result, already acceptable, was further improved with the selection of the informative variables by means of GA. In particular, the retained explanatory variables were 1 milk alkane (C24) and 10 milk FA concentrations, classes, or ratios: C13:0 *iso*, C16:1 7c, C18:1 12c, C18:2 9c 12c, C20:0, CLA11t 13t, C20:3 5c 8c 11c, C20:5 5c 8c 11c 14c 17c, and n6, n3/n6. The model had an RMSECV% of 7.4% and an explained variance of 80.8%. Figure 2B shows the cross-validated vs. the experimental value plot. The former data were obtained in the prediction of HP using the selected variables.

## Discussion

The discrimination of milk sourced from mixed diets of ewes with ATs to pasture of 2, 4, or 6 h/d performed better than the discrimination of cow milk from animals fed fresh forages (>50% DM from fresh forage) or mixed diets [diets in which none of the forages under scrutiny–fresh forage, grass silage, and corn silage–reached at least half of the dietary DM, (19)]. In that case, the milk samples were correctly classified in 84% of cases with fresh forages and in 57.6% of cases with mixed diets. In contrast, results of this note are similar to those based on the analysis of visible and near-infrared spectra of milk samples by Coppa et al. (20): in that case, the error of classification of pasture-based compared with hay-based diets was only 8.5%.

The FAs most relevant for the discrimination (C13:0 *iso*, C14:0, C16:1 7c, C18:1 11t, CLA 11t 13c, C22:5 7c 10c 13c 16c 19c) partially align with those found by Coppa et al. (18) as biomarkers of fresh forages in milk of dairy cows. In our case, an important role was played by some FAs sourced from *de novo* synthesis (C13:0 and C14:0) or from mixed origin (C16:1 7c). According to Vlaminck et al. (21), odd-chain FAs, such as C13:0 and C17:0, are potential markers of microbial activity, assuming their ruminal origin. In contrast, long-chain FAs, such as C18:1 11t and CLA 11t 13c, are in line with previous results in sheep (22) and goats (23) that showed higher levels in milk sourced from fresh herbage-based diets. The higher polyunsaturated n−3 FA concentration in the diet richest in grazed herbage (6 h/d group, Table 1) explains the presence also of C22:5 7c 10c 13c 16c 19c (DPA), a long-chain n−3 polyunsaturated fatty acid (PUFA) that comes from the elongation of α-linolenic acid.

The only alkane selected by GA for milk authentication was C24 in milk, which was also the shortest chain alkane detected in milk. Its amount and its proportion on total milk alkanes are both low (1.08 mg/L and 4.08%), with numerically higher levels in milks of sheep with the lowest allocation to pasture (1.33, 0.86, and 1.06 mg/L in 2, 4, and 6 h/d treatment groups). Although grasses contain mainly long-chain alkanes (C29–C33, Supplementary Table 1), their digestibility is low, and this explains their low milk concentration in our milk dataset. The level of C24 was almost undetectable in feces, confirming its probable digestion and uptake. To the best of our knowledge, data on n-alkane concentration in sheep milk are not available; hence, this finding warrants further investigation.

The estimation of herbage intake was moderately precise after the selection of the relevant variables. The main explanatory variables are overall related to the level of PUFA in sheep diet and hence in milk, particularly n−3 FA, such as C18:3 9c 12c 15c, in the herbage (CLA 11t 13t, C20:5 5c 8c 11c 14c 17c, and n3/n6) and to the ruminal metabolism of amino acids (C13:0 iso) (21). Another FA, C20:0, was found to be moderately negatively correlated to dietary herbage contribution (*r* = −0.49, *P* < 0.05), and it could be possibly associated with the intake of lupin seed (see below).

The estimation of the proportion of herbage in the diet after GA-LDA was similar to that obtained by Coppa et al. (19) in bulk cow milk samples gathered across Europe and submitted to conventional FA analysis (R^2^ = 0.81 in calibration and 0.79 in validation). In our study, as expected, the most relevant biomarkers for the prediction of HP were partially the same selected for the prediction of HDMI. They were the alkane in milk C24 and the FAs C13:0 iso, C16:1 7c, C20:0, CLA11t 13t, C20:5 5c 8c 11c 14c 17c, and n3/n6. The other FA estimators, such as C18:2 9c 12c and C20:3 5c 8c 11c, are possibly metabolites sourced from lupin seed intake. Linoleic acid, together with oleic acid (Supplementary Table 1), is the most relevant FA in the lupin seed used in our study, and C20:3 5c 8c 11c could derive from the metabolism of linoleic acid by the elongase/desaturase activities that occur at the mammary level.

## Conclusion

This note shows that using milk FA and n-alkane to trace milks from dairy sheep submitted to PTG provides encouraging results. Firstly, the authentication performance based on one milk n-alkane and six milk FAs as biomarkers was very good, with almost 96% of samples correctly classified. Fecal alkanes were not selected as biomarkers of feeding regimen. This means that the combined use of FAs and alkane biomarkers in milk allows to successfully distinguish milks that come from pretty similar mixed feeding regimens, differing for only 2 h/d AT to pasture. Secondly, this study shows that the proportion of herbage in sheep diet can be precisely estimated using the above biomarkers. Finally, herbage intake can also be predicted, although estimates are only moderately precise. These results need to be confirmed on a longer grazing period using a wider database, possibly including other forage species and different supplementation levels.

## Data Availability Statement

The raw data supporting the conclusions of this article will be made available by the authors, without undue reservation.

## Ethics Statement

The animal study was reviewed and approved by Agris ethical committee.

## Author Contributions

GM planned the experiment with AC and MAd and contributed to the on-field data collection and to the writing of the manuscript. MD and MAc contributed to the on-field experimental setting. GS and MF carried out the n-alkanes and FA analyses, respectively. MC carried out the statistical analysis and contributed to the writing of the paper. AC and MAd edited a preliminary release of the paper. All authors contributed to the last edition.

## Conflict of Interest

The authors declare that the research was conducted in the absence of any commercial or financial relationships that could be construed as a potential conflict of interest.
