# Supplementary material for: A Note on the Tracing of Herbage Contribution to Grazing Sheep Diet Using Milk and Feces Biomarkers
Source: Front Vet Sci. 2021 Feb 19;8:623784. doi: 10.3389/fvets.2021.623784 (PMC7933569; doi:10.3389/fvets.2021.623784)
Supplement: Supplementary file 1 [file Table_1.docx]

Supplementary table 1. Feedstuff composition (mean and standard error)

|  | **Herbage 2 h/d** | | **Herbage 4 h/d** | | **Herbage 6 h/d** | | **Hay^#^** | | **Lupin^#^** | | **Concentrate^#^** | |
| --- | --- | --- | --- | --- | --- | --- | --- | --- | --- | --- | --- | --- |
|  | Mean | SE | Mean | SE | Mean | SE | Mean | SE | Mean | SE | Mean | SE |
| **Nutrients**  **(g/kg DM)** |  |  |  |  |  |  |  |  |  |  |  |  |
| DM | 192.1 | 9.2 | 192.7 | 0.7 | 198.8 | 8.7 | 858.5 | 1.7 | 903.3 | 4.7 | 878.4 | 7.4 |
| EE | 39.6 | 2.1 | 38.8 | 0.0 | 38.0 | 1.3 | 21.7 | 1.6 | 57.8 | 2.1 | 28.2 | 2.1 |
| CP | 139.2 | 18.5 | 137.5 | 7.2 | 142.3 | 6.1 | 102.3 | 2.3 | 334.6 | 1.1 | 169.9 | 2.5 |
| Ash | 114.0 | 3.5 | 115.8 | 1.4 | 118.9 | 8.1 | 77.4 | 0.3 | 31.8 | 1.2 | 129.8 | 1.8 |
| NFC | 260.4 | 38.2 | 264.8 | 19.7 | 240.8 | 8.6 | 199.5 | 0.8 | 288.9 | 22.3 | 309.2 | 15.3 |
| NDF | 446.7 | 14.2 | 443.1 | 11.1 | 460.0 | 7.9 | 599.3 | 2.8 | 307.0 | 24.3 | 363.1 | 6.9 |
| ADF | 222.3 | 6.6 | 222.1 | 6.1 | 227.8 | 7.7 | 388.3 | 1.8 | 197.1 | 2.7 | 193.6 | 11.6 |
| ADL | 5.7 | 3.1 | 4.4 | 1.1 | 5.7 | 3.2 | 58.0 | 0.4 | 6.0 | 1.0 | 34.2 | 0.6 |
| IVDMD | 869.5 | 5.7 | 877.3 | 10.5 | 853.9 | 7.5 | 565.6 | 9.4 | 920.1 | 6.6 | 768.3 | 5.2 |
|  |  |  |  |  |  |  |  |  |  |  |  |  |
| **FAME**  **(g/kg DM)** |  |  |  |  |  |  |  |  |  |  |  |  |
| C4:0 | 0.13 | 0.05 | 0.10 | 0.04 | 0.13 | 0.03 | 0.03 |  | 0.01 |  | 0.01 |  |
| C6:0 | 0.02 | 0.01 | 0.03 | 0.01 | 0.03 | 0.01 | 0.06 |  | 0.02 |  | 0.43 |  |
| C7:0 | 0.03 | 0.00 | 0.03 | 0.00 | 0.03 | 0.00 | 0.05 |  | 0.04 |  | 0.04 |  |
| C8:0 | 0.01 | 0.00 | 0.01 | 0.00 | 0.01 | 0.00 | 0.02 |  | 0.02 |  | 0.02 |  |
| C10:0 | 0.01 | 0.01 | 0.01 | 0.00 | 0.01 | 0.00 | 0.02 |  | 0.02 |  | 0.01 |  |
| C11:0 | 0.11 | 0.00 | 0.15 | 0.02 | 0.20 | 0.03 | 0.01 |  | 0.01 |  | nd |  |
| C12:0 | 0.06 | 0.00 | 0.07 | 0.01 | 0.08 | 0.01 | 0.06 |  | 0.03 |  | 0.02 |  |
| C13:0 i | 0.05 | 0.00 | 0.07 | 0.01 | 0.09 | 0.03 | 0.03 |  | 0.02 |  | nd |  |
| C13:0 ai | 0.08 | 0.01 | 0.11 | 0.02 | 0.15 | 0.02 | 0.02 |  | 0.01 |  | 0.01 |  |
| C14:0 | 0.08 | 0.01 | 0.08 | 0.00 | 0.09 | 0.01 | 0.15 |  | 0.12 |  | 0.08 |  |
| C14:1 9c | 0.01 | 0.00 | 0.01 | 0.00 | 0.02 | 0.00 | 0.05 |  | 0.04 |  | 0.04 |  |
| C15:0 | 0.08 | 0.02 | 0.12 | 0.00 | 0.16 | 0.02 | 0.02 |  | 0.01 |  | 0.01 |  |
| C16:0 | 2.43 | 0.27 | 2.72 | 0.11 | 3.36 | 0.21 | 2.33 |  | 5.58 |  | 4.62 |  |
| C16:1 7c | 0.26 | 0.10 | 0.39 | 0.01 | 0.53 | 0.03 | 0.06 |  | 0.03 |  | 0.02 |  |
| C16:1 9c | 0.02 | 0.00 | 0.01 | 0.00 | 0.02 | 0.01 | 0.01 |  | 0.00 |  | 0.01 |  |
| C17:0 | 0.03 | 0.00 | 0.03 | 0.00 | 0.04 | 0.00 | 0.05 |  | 0.04 |  | 0.04 |  |
| C18:0 | 0.20 | 0.01 | 0.20 | 0.00 | 0.21 | 0.02 | 0.29 |  | 2.53 |  | 0.50 |  |
| C18:1 9c | 0.19 | 0.04 | 0.21 | 0.01 | 0.24 | 0.01 | nd |  | 13.42 |  | 3.08 |  |
| C18:1 11c | 0.02 | 0.00 | 0.03 | 0.00 | 0.03 | 0.00 | 0.03 |  | 0.36 |  | 0.16 |  |
| C18:2 9c,12c n6 | 0.57 | 0.01 | 0.81 | 0.01 | 1.01 | 0.13 | 0.92 |  | 14.82 |  | 2.64 |  |
| C18:3 9c,12c,15c n3 | 1.81 | 0.18 | 3.16 | 0.42 | 4.40 | 0.80 | 0.18 |  | 1.76 |  | 0.15 |  |
| C20:0 | 0.07 | 0.00 | 0.06 | 0.01 | 0.07 | 0.00 | 0.12 |  | 0.32 |  | 0.12 |  |
| C20:4 5c,8c,11c,14c n6 | 0.01 | 0.00 | 0.01 | 0.00 | 0.01 | 0.00 | 0.04 |  | 0.04 |  | 0.03 |  |
| C21:0 | 0.13 | 0.01 | 0.11 | 0.01 | 0.13 | 0.01 | 0.02 |  | 0.03 |  | nd |  |
| C22:0 | 0.13 | 0.01 | 0.09 | nd | 0.15 | 0.02 | 0.17 |  | 0.66 |  | 0.11 |  |
| C22:6 4c,7c, 10c,13c,16c,19c n3 | 0.03 | 0.02 | 0.05 | 0.00 | 0.06 | 0.00 | 0.01 |  | 0.01 |  | 0.02 |  |
| C23:0 | 0.18 | 0.00 | 0.15 | 0.01 | 0.17 | 0.02 | 0.08 |  | nd |  | 0.03 |  |
| C24:0 | 0.06 | 0.01 | 0.06 | 0.00 | 0.07 | 0.01 | 0.09 |  | 0.11 |  | 0.09 |  |
| C26:0 | 0.08 | 0.03 | 0.09 | 0.00 | 0.09 | 0.00 | 0.06 |  | 0.01 |  | 0.05 |  |
|  |  |  |  |  |  |  |  |  |  |  |  |  |
| **N-alkanes* (mg/kg DM)** |  |  |  |  |  |  |  |  |  |  |  |  |
| C24 | 1.10 | 0.31 | 1.16 | 0.06 | 0.86 | 0.09 | nd | nd | 0.34 | 0.02 | 0.55 | 0.04 |
| C25 | 10.09 | 0.63 | 9.90 | 0.91 | 10.59 | 1.32 | 17.28 | 0.46 | 3.03 | 1.21 | 7.34 | 0.26 |
| C26 | 1.56 | 0.80 | 1.32 | 0.23 | 0.87 | 0.06 | 1.44 | 0.08 | 0.30 | 0.02 | 0.74 | 0.17 |
| C27 | 35.16 | 1.40 | 34.33 | 0.20 | 34.84 | 1.45 | 36.61 | 1.52 | 1.48 | 0.20 | 12.61 | 1.81 |
| C28 | 6.88 | 0.21 | 9.05 | 0.67 | 7.45 | 0.06 | 9.27 | 4.08 | 2.83 | 1.20 | 3.40 | 0.75 |
| C29 | 205.96 | 3.66 | 201.57 | 1.46 | 203.05 | 3.03 | 102.41 | 4.54 | 3.60 | 0.58 | 59.21 | 2.97 |
| C30 | 10.20 | 0.98 | 10.21 | 0.95 | 10.40 | 1.21 | 5.10 | 0.31 | 0.10 | nd | 1.91 | 0.43 |
| C31 | 299.96 | 1.36 | 292.62 | 1.12 | 293.53 | 2.11 | 163.92 | 4.62 | 1.53 | 0.47 | 54.78 | 14.09 |
| C32 | 3.67 | 0.43 | 3.63 | 0.12 | 3.70 | 0.42 | 2.92 | 0.09 | 0.00 | nd | 1.13 | 0.29 |
| C33 | 67.92 | 0.34 | 63.84 | 1.21 | 66.78 | 2.48 | 13.49 | 0.59 | 0.24 | nd | 6.46 | 0.87 |

# Bulked samples results for FA analysis; nd = not detectable; * n-alkanes with chain > C33 were nd.
