# Supplementary material for: A Note on the Tracing of Herbage Contribution to Grazing Sheep Diet Using Milk and Feces Biomarkers
Source: Front Vet Sci. 2021 Feb 19;8:623784. doi: 10.3389/fvets.2021.623784 (PMC7933569; doi:10.3389/fvets.2021.623784)
Supplement: Supplementary file 2 [file Data_Sheet_1.docx]

*Milk n-alkane analysis*

*Reagents*

ACS ISO grade ethanol 95%, hexane Laboratory Reagent (purity > 95%), potassium hydroxide (for analysis, ACS-ISO) were from Carlo Erba Reagents, Milan, Italy. Standard n-alkanes from C23 to C36 were obtained from Sigma Aldrich (Milan, Italy).

*Analytical procedure*

The n-alkane analysis of herbage and feces followed the protocol by Dove and Mayes (2006). A similar protocol was implemented for milk alkane determination. In particular, 0.4 g of milk fat (0.5 g DM of feces) in a screw cap tube is weighed and 0.06 mg (0.15 mg for feces) of C34 as internal standard and 10 ml (5 ml for feces) of an ethanolic solution of KOH (5% (w/v) of KOH in 95% ethyl alcohol) are added. The test tube is incubated overnight at 90 °C. After cooling, 4 ml (2 ml for feces) of H_2_O and 10 m (5 ml for feces) of n-hexane are added and the test tube is heated at 60 °C, stirred and the phases are separated. The organic phase is recovered in another test tube, the aqueous phase is washed 2 times with 5 ml of hexane, always recovering in the same test tube the organic phases. Solvent is evaporated to dryness in a rotavapor and the residue taken up with 1 ml of n-hexane. This organic solution is further purified on a 10 cm column prepared with 2 g of silica gel for chromatography (0.063-0.200 mm) and previously balanced with n-hexane; alkanes are eluted with about 10 ml of n-hexane. The solvent is evaporated to dryness and residue taken up with 100 µl (1 ml for feces) of n-eptane. Finally, 1 microliter is injected into the gas chromatograph and analyzed.

The quantitative determination of alkanes profile of milk samples was accomplished on a Dani Master GC (Dani Analitica, Milan, Italy) equipped with a SPB tm-1 (Supelco Inc.) capillary column (30 m length, 0.53 mm i.d., 0.1 µm film tickness), a PTV injector and a Flame Ionization Detector (FID) and controlled by the software Clarity (Dani Analitica, Milan, Italy). The operating chromatographic conditions were: carrier gas: helium; flow: 1 ml min^-1^; initial temperature of PTV injector 50 °C for 0.17 min and final temperature 290 °C for 15 min.; oven temperature program: 2 min at 200 °C, then from 200°C to 290 °C at a rate of 12°C min^-1^, 290 °C isotherm for 3.5 min; FID temperature: 340 C.

Individual n-alkanes from C23 to C36 were identified by the comparison of the retention time of a standard mixture of pure components.

Furthermore, indices were calculated with reference to n-alkane such as the ratio between the concentrations of adjacent alkanes with carbon chain length ranging between C27 and C33 (C27/C25, C29/C27, C31/C29, C33/C31).
